# Seasonal diversity, daily use and behaviour of birds using a green roof in a Mediterranean city

**DOI:** 10.1098/rsos.240761

**Published:** 2024-12-04

**Authors:** Álvaro Luna, Estel Blanch

**Affiliations:** ^1^ Department of Biosciences, Faculty of Biomedical and Health Sciences, Universidad Europea de Madrid, Madrid, 28670, Spain; ^2^ BiBio Research Group, Natural Sciences Museum of Granollers, Granollers 08402, Spain

**Keywords:** urban ecology, *Columba*, Europe, urban design, sustainable architecture

## Abstract

Urban green roofs offer environmental and social benefits and provide resources for urban wildlife; however, how birds use green roofs remains poorly studied in Mediterranean cities. Here, we develop a 1-year study in Madrid, Spain, recording the birds that use both an urban green roof and the adjacent conventional roofs throughout the four seasons. We recorded a total of 17 bird species in the area, of which 8 use the green roof surveyed. The most common species detected in both types of roofs was the feral pigeon (*Columba livia*), which exploited the green roof permanently, in contrast with the other species observed, which only appeared occasionally. We also found that more species visited the green roof in the central hours of the day and a higher richness of species in the spring. Moreover, we detect that most of the species were residents all year in the area and have granivorous or granivorous–omnivorous feeding habits. The main behaviour observed in the green roof was feeding, while no reproduction of any species was confirmed. We conclude that green roofs offer birds much-needed resources in metropolitan areas, promoting greener, more connected and more biodiverse cities.

## 1. Introduction

The human migration to urban areas is occurring at an unprecedented rate. While one century ago approximately 10% of humans inhabited cities, at this moment about 50% did so, with 70% predicted to live in urbanized landscapes by 2050 [1]. Urban sprawl is one of the most prevalent causes of landscape transformation worldwide, creating new habitats starkly different from the natural habitats it replaces [2]. In general, the urbanization process results in a loss of biodiversity through local extinction and homogenization of bird communities [3,4], but in recent years, multiple approaches have emerged to make cities more sustainable and favourable for a greater diversity of species [5]. Today it is considered that, among birds, nearly 20% of the approximately 10 000 described species can be found in cities [6], a habitat that sometimes hosts populations of endangered species [7–10]. Within cities, their green areas are key for biodiversity conservation because they constitute refuges that enable certain native taxa to colonize and persist in urban environments [11,12]. In this context, emerging recognition of the benefits associated with green infrastructures has led to a major interest towards the creation of green roofs in cities as a solution to use the available space in buildings [13].

Among the positive effects, urban green roofs have thermal benefits, including improving local microclimate, retaining storm water, reducing the urban heat island effect and air pollution, making cities more resilient to climate change, producing food and providing aesthetic benefits [13–17]. From a biodiversity perspective, some features of the green roofs play a determinant role that determines the presence of animals and plants: the height of the building, the distance to the ground and the distance to the nearest vegetated patches and its accessibility, the vegetation cover, the age and size of the green roof, and the presence or absence of suitable habitats [18–20]. In general, green roofs can potentially provide habitat for many species in urban environments, and they can act as corridors and stepping stones for urban plants and animals as they connect parks and gardens in densely inhabited cities [21–23]. Moreover, urban green roofs may contribute to maintain populations of pollinators in cities, as they can exploit them for food, refuge and breeding, as demonstrated for bees [24] and butterflies [25]. They can also be a habitat that fungi [26], mosses, ferns and spermatophytes spontaneously colonize [27–29] and offer a habitat for urban bats [30].

In the last decades, different studies have assessed the presence of birds on green roofs around the world. Today we know that they use green roofs for food and water, shelter, nesting and resting [31]. As an example, Deng & Jim [28] showed that 10 resident bird species and 6 migrant winter visitors use a 1000 m^2^ green roof in Hong Kong city. In the USA, Coffman & Waite [32] detected eight species on green roofs located in the Great Lakes and Eastern Corn Belt Plains. Moreover, Wang *et al*. [25] reported the occurrence of 53 bird species on 30 urban green roofs in Singapore, observing reproductive behaviours in 20 of the species cited. Regarding breeding, Duncan *et al*. [33] showed the role that roofs play in the reproduction of Eurasian oystercatchers (*Haematopus ostralegus*) in Aberdeen (UK). Similarly, Baumann [34] reported the breeding of the little ringed plover (*Charadrius dubius*) and northern lapwing (*Vanellus vanellus*) on green roofs in Switzerland. However, we still lack evidence on the use of green roofs by birds in Mediterranean cities, despite the Mediterranean basin being considered a hot spot of biodiversity [35]. Here, we conduct a 1-year study of the birds using a green roof located in the core of Madrid (Spain) and the surrounding conventional roofs to (i) measure differences in the richness and the species composition in both substrates in the four seasons and different periods of the day; (ii) classify how the birds use the green roof according to their diet preferences; and (iii) determine the observed behaviour to ascertain the predominant use of the green roof under study by the birds.

## 2. Material and methods

### 2.1. Study area

We developed our study on a green roof that covers 75% of the top of Fundación Biodiversidad headquarters in Madrid. It is composed of a layer of 5 cm of substrate with a water channelling system to collect both irrigation leachate and rainwater, later stored for reuse both for irrigation and other uses of the building (Fundación Biodiversidad, personal communication). Originally, 10% of the surface was planted with *Sedum album* and 40% with *Sedum acre*, with a planting density of approximately 25 plants m^−^². Currently, these two species cover almost 100% of the surface, together with spontaneous unidentified grasses and moss, but with no shrubs and trees (personal observation). The green roof is surrounded by taller residential buildings, so it is about 20 m below the level of the adjacent roofs.

The green roof is located in a densely inhabited district (235.16 inhabitants/hectare [36]) in Madrid, central Spain (40.42° N, 3.70° W). The city is in a region with a Mediterranean climate (Csa Köppen-Geiger climatic classification [37]), with hot summers and cold winters, and precipitation mostly concentrated in spring and autumn. The neighbourhood has tree-lined avenues and streets, mainly composed of London planes (*Platanus × hispanica*), honeyberries (*Celtis australis*), broad-leaf privet (*Ligustrum lucidum*) and Japanese pagoda trees (*Styphnolobium japonicum*) [38]. The nearest parks and gardens are the Casino de la Reina Park (at approx. 200 m; approx. 2 ha), Peñuelas Park (at approx. 300 m; approx. 3.5 ha), Madrid Río Park (at approx. 700 m; approx. 40 ha) and the Royal Botanical Garden (at approx. 1200 m; approx. 7.4 ha).

### 2.2. Study design

We divided the observations into 30 min sessions, covering simultaneously both the green roof (623 m^2^) and the surrounding buildings with conventional roofs made of clay roof tiles (450 m^2^). We conducted our observations during 28 days per season, during four consecutive seasons (Autumn 2021, Winter 2021–2022, Spring 2022, Summer 2022). For each day, we conducted the observations in one of the four pre-established periods (1: first 2 hours after sunrise; 2: 10.00–12.00; 3: 13.00–15.00; 4: last 2 hours before sunset). Each observation is retained as a replicate, resulting in seven replicates per period and season, so a total of 112 replicates for the green roof and 112 replicates for the surrounding buildings with conventional roofs. With slight modifications, we followed Deng & Jim [28] to classify the bird species observed by residence status in the area, ordered by residents and migrants (this category includes spring to summer and winter visitors, as well as passage migrants), and by feeding preferences (granivores, insectivores, insectivore–granivores, insectivore–frugivores). In this sense, we also included two additional categories, predators (here used when the species include vertebrates in the diet, to differentiate from the insectivorous category) and scavengers, given their presence in cities [12], and because some of the potential species occurring in the study area have opportunistic habits and can consume both plant-based items and live or dead animals. To check this information, we used the III Spanish Breeding Bird Atlas (https://atlasaves.seo.org/; [39]). We also recorded the behaviour observed in the birds occurring in both kinds of roofs, classifying them into three categories: feeding, resting and breeding. The observation point was always the same window located on the top floor of one of the buildings, with full vision of the green roof and almost 360° vision of the surrounding building roofs. Although the distance to the green roof and the other buildings is close, we recorded the bird species and their behaviour with binoculars. We initially considered the presence of bird feeders and sources of water as a potential artefact that could influence our observations [40,41], but during the study, we never observed neighbours feeding the birds, and no bird feeders and sources of water were installed in the windows and balconies.

### 2.3. Statistical approach

We used generalized linear models to assess the effects of the buildings (birds using the green roof versus birds using the surrounding buildings with conventional roofs) and environmental variables (season of the year and pre-established period) on the bird richness observed (Poisson error distribution and ‘log’ link function). Non-overdispersion was confirmed using the *gof* function, R package aods3 [42]. We also checked no multi-collinearity in the explanatory variables by calculating the variance inflation factors (VIF) with the *vif* function, R package car [43]. Our variables had a VIF < 4 [44].

From the saturated model, which also includes the interactions between the variables, we used the *dredge* function (R package MuMIn [45]) to generate a complete set of models. Model selection was performed using the Akaike information criterion (AICc) corrected for small sample sizes. The best AIC model was the final selected model, as it was also the only one within 2 AIC units (table 1). Finally, to assess the effect of each factor, we conducted a Tukey post hoc test (R package emmeans [46]).

**Table 1.** Models obtained to assess the relative importance of the site (green roof versus surrounding buildings with conventional roofs) and environmental variables (season of the year and pre-established periods) on the bird richness observed. Models shown are the first 10 models ranked using their AICc. ΔAICc, difference in AICc of each model compared with the best model (the model with the lowest AICc); AIC, Akaike information criterion; d.f., degrees of freedom; W, Akaike weights. In bold, the model selected corresponds to the best model (lowest AICc).

model	d.f.	AICc	ΔAICc	W
**season + site ∗ period**	**11**	**606.16**	**0**	**0.62**
site ∗ period + site ∗ season	14	608.49	2.33	0.19
site + season + period	8	609.92	3.75	0.09
site + season	5	611.63	5.46	0.04
period + site ∗ season	11	612.05	5.89	0.03
site ∗ season	8	613.58	7.42	0.02
site ∗ period + period ∗ season	20	616.31	10.15	<0.01
season + period	7	616.70	10.53	<0.01
season	4	618.47	12.30	<0.01
site + period ∗ season	17	619.47	13.31	<0.01

We also tested for differences in the species composition among the studied infrastructures and environmental variables. For this, we used a permutational multivariate analysis of variance (PERMANOVA [47]). The model was generated with a Jaccard distance matrix, based on the presence or absence of each species, using 9999 random permutations with the *adonis* function (vegan R package [48]). The PERMANOVA results were only considered when the variance of the group was homogeneous, which was tested with the *betadisper* function (R package vegan [48]). The results were visualized using principal coordinates analysis (PCoA). We also conducted an analysis of similarity percentages (*simper* function; R package vegan [48]) to assess the exact species that significantly differ in frequency of occurrence (hereafter FOO) between groups. Statistical analyses were conducted in R 4.1.1 [49].

## 3. Results

**Figure 1.**
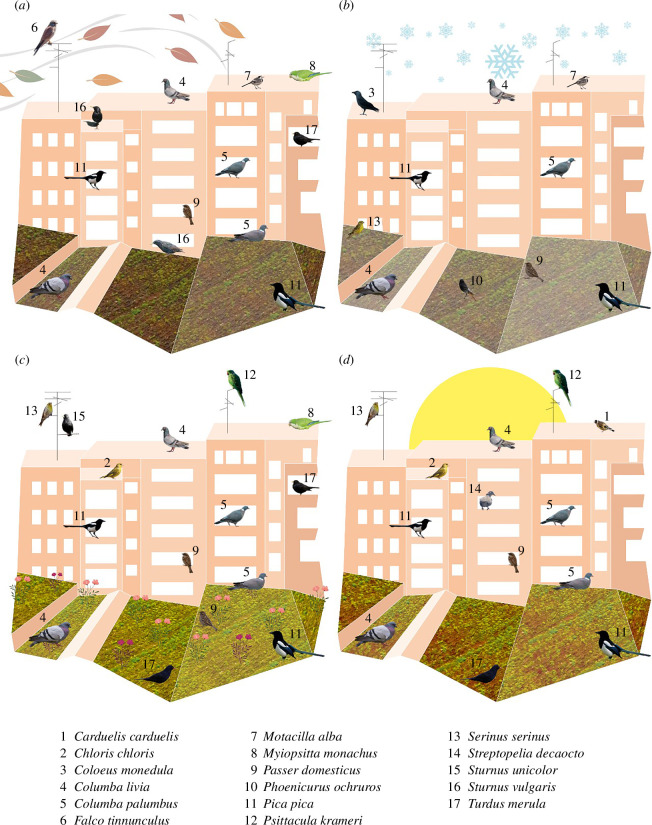
Bird species observed on both green roofs and surrounding buildings with conventional roofs across the autumn (*a*), the winter (*b*), the spring (*c*) and the summer (*d*).

We recorded 17 bird species in the study area (figure 1): *Carduelis carduelis*, *Chloris chloris*, *Coloeus monedula*, *Columba livia*, *Columba palumbus*, *Falco tinnunculus*, *Motacilla alba*, *Myiopsitta monachus*, *Passer domesticus*, *Pica pica*, *Phoenicurus ochruros*, *Psittacula krameri*, *Serinus serinus*, *Streptopelia decaocto*, *Sturnus unicolor*, *Sturnus vulgaris* and *Turdus merula*. Eight species were detected on the green roof and 16 species on the surrounding conventional roofs. *Phoenicurus ochruros* was the only species detected exclusively on the green roof, while *C. carduelis*, *C. chloris*, *C. monedula*, *F. tinnunculus*, *M. alba*, *M. monachus*, *P. krameri*, *S. decaocto* and *S. unicolor* were detected on the surrounding conventional rooftops but not on the green roof (figure 1). *Columba livia* was the most reported species, occurring on 95.5% of the days on conventional roofs and 92% on the green roof. This species was followed by the common wood pigeon (*C. palumbus*), the blackbird (*T. merula*), the Eurasian magpie (*P. pica*) and the house sparrow (*P. domesticus*) (electronic supplementary material, SP1).

## 4. Green roof versus conventional roofs: seasonal and daily use

**Figure 2.**
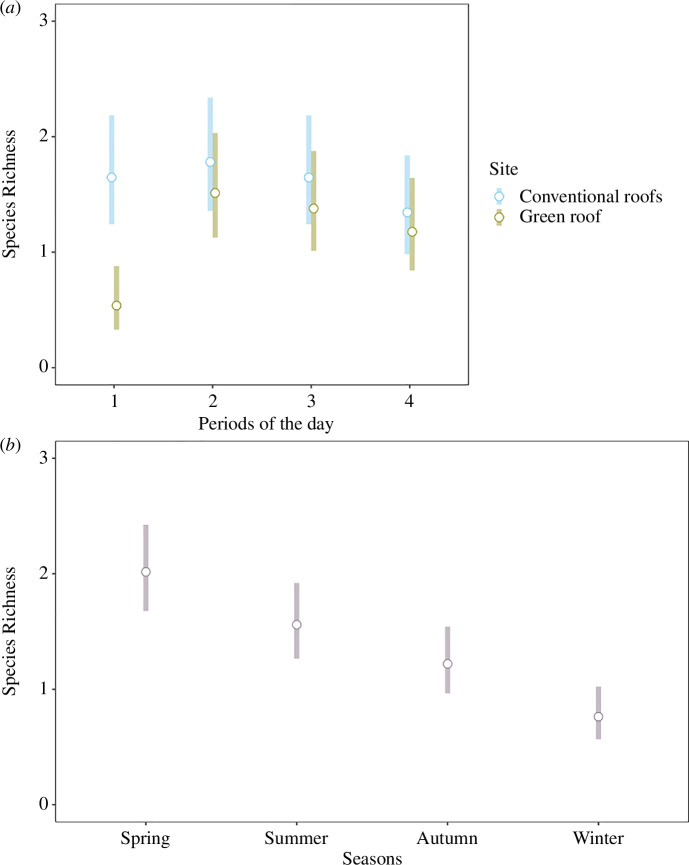
*(a*) Bird species richness in the green roof (green) and the adjacent conventional roofs (blue) along the pre-established periods of the day. (*b*) Bird species richness along the seasons (including conventional and green roofs), classified in decrescent order. Bars represent the 95% confidence intervals.

The species richness was found to be significantly influenced by the interaction between the site and the period of the day and by the season (table 2). We observed higher species richness in the conventional roofs than in the studied green roof in the first period of the day (first 2 hours after sunrise) (figure 2*a*). Indeed, although we did not observe significant differences in the conventional roofs during the different periods of the day (1.78 ± 0.25 to 1.34 ± 0.21), in the green roof the species richness was significantly lower (0.53 ± 0.13) during the first hours after the sunrise than in the rest of the day, which fluctuated from 1.18 ± 0.20 to 1.51 ± 0.23 (figure 2*a*, electronic supplementary material, SP2). We also detected a higher richness in both types of roofs in the spring in comparison with the winter and the autumn (*p *< 0.05, figures 1, 2b, electronic supplementary material, SP2).

**Table 2.** Results of the generalized linear model conducted with Poisson error distribution testing the effect of the site (green roof versus surrounding buildings with conventional roofs) and environmental variables (season of the year and pre-established periods) on the bird richness observed. Significant *p*-values (*p *< 0.05) are highlighted in bold.

variable	estimate	s.e.	*z*-value	*p*‐value
(intercept)	0.932	0.161	5.807	<0.001
site: green roof	−1.119	0.288	−3.887	**<0.001**
period: 2	0.078	0.198	0.396	0.692
period: 3	<0.001	0.202	<0.001	1.000
period: 4	−0.203	0.213	−0.952	0.341
season: summer	−0.257	0.139	−1.854	0.064
season: autumn	−0.502	0.149	−3.365	**0.001**
season: winter	−0.972	0.175	−5.557	**<0.001**
site: green roof ∗ period: 2	0.956	0.352	2.714	**0.007**
site: green roof ∗ period: 3	0.941	0.357	2.633	**0.008**
site: green roof ∗ period: 4	0.986	0.369	2.668	**0.008**

**Figure 3.**
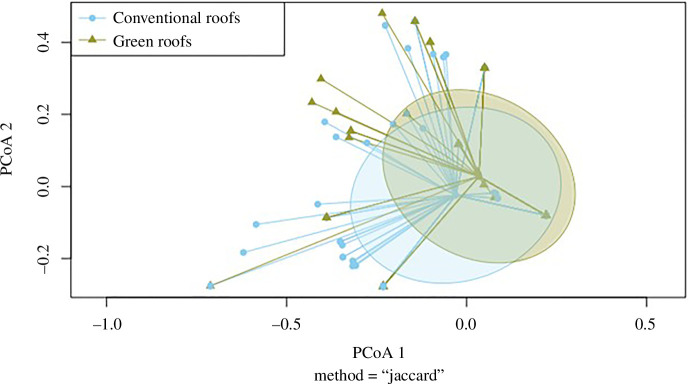
Variance in the bird species found in the conventional roofs and the green roof for the 112 surveys conducted in each site. Represented by the first two axes of a principal coordinates analysis (PCoA), using Jaccard distance matrix for presence-absence data. The individual points represent the data points for each site (conventional roofs are dots, and green roofs are triangles). The centroid represents the average position of the data points for that group. The ellipses represent the dispersion of the data around that centroid within a standard deviation.

Figure 3 represents the composition of birds found in both sites, and it shows a very similar dispersion between the conventional roofs and the green roof, as both areas are very similar. This approach allows us to trust the PERMANOVA result that expresses a significant difference in bird species composition between both studied roof types (electronic supplementary material, SP3). Although it could be considered that the nine species present only in conventional roofs are responsible for this difference, those birds occur at a very low frequency, from 1% to 4%. Thus, the analysis showed that the main species responsible for the dissimilarity between conventional and green roofs are *C. palumbus* and *P. ochruros* (electronic supplementary material, SP4). This is explained because *C. palumbus* was found on conventional roofs more frequently than on the green roof (32% and 14% FOO, respectively), and *P. ochruros* was the only species not found on conventional roofs, occurring 7% of the time on the green roof.

## 5. Residence status, diet preferences and behaviour

**Figure 4.**
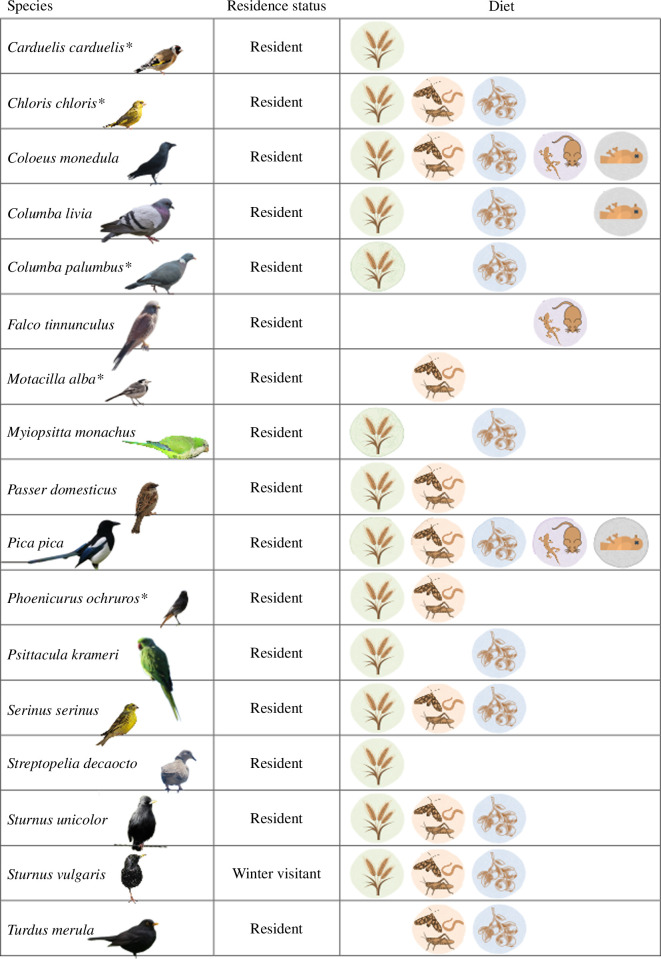
The residence status and diet of the species observed on both green and conventional roofs during our study. * Spain receives a contingent of birds from Europe during winter or from Africa/Europe during the migratory passage, who can join temporarily the permanent population. From left to right, the diet figures correspond to granivore, insectivore, frugivore, carnivore and scavenger.

We found that almost all the species observed are residents of Madrid, except the common starling (*S. vulgaris*), which at the latitude of Madrid is essentially a winter bird. Regarding diet, most of the species cited in the area have an omnivorous diet, consuming plant-based resources but also invertebrates (especially during the breeding season) and carrion (figure 4). Only the Eurasian kestrel (*F. tinnunculus*) can be considered a strict predator among the species detected, while the white wagtail (*M. alba*) is essentially insectivorous. Finally, regarding the behaviours observed, on the green roof, all the observations have been birds feeding or foraging in search of food, while no birds resting or using the area for reproductive purposes were detected. On the contrary, birds observed on conventional roofs basically used it to rest (looking for the sun in winter and the shadow in the summer) and sleep, with the occasional observation of some feral pigeons mating.

## 6. Discussion

In this study, we explored the diversity of birds observed during 1 year on a green roof and its surrounding conventional roofs. Our results confirm the presence of 8 species that use the green roof and 16 observed in the adjacent buildings, with a higher species richness in the spring and a lower number of species using the green roof in the first hours after sunrise. Regarding the low number of species in comparison with other studies [25,28], we must consider the species occurring in the area that potentially can use the green roof. Moreover, we think that the location of a green roof, in a densely populated neighbourhood and below the level of the surrounding buildings, can reduce its detectability by birds or could cause mistrust in birds perched or flying closer. Moreover, this location can have as a consequence a low level of sunlight in the first hours of the day, which may explain the less use that birds make of it in this period of the day. On the other hand, the limited supply of resources, as the green roof is mainly based on *Sedum*, may not be attractive to a larger number of birds.

Among the species recorded, the most common was the feral pigeon, with a peak of records in summer, while the presence of most species reported is anecdotal, with less than five observations in the studied year. The feral pigeons have a plant-based diet, but in cities, they are subsidized with human-related food resources, which contributes to increasing their density [50], and the human–pigeons conflict and bad perception towards the species [51]. The presence of green areas within the urban matrix such as green roofs offers diet options less dependent on human discards. This, along with active awareness campaigns not to feed the pigeons and effective cleaning services in the city, could contribute to a natural reduction in feral pigeon abundance through a potential decrease in the number of broods [52,53].

Most of the species recorded were resident in the studied area. However, in some cases, the populations of some species can increase with the arrival of migrating individuals wintering in Spain. Almost all the behaviours observed in the green roof by the different species were related to food acquisition, with individuals foraging in the green roof for seeds and other plant-based resources. Although we adapted Deng & Jim [28] to include predators and scavengers, we only noted anecdotally the presence of a common kestrel (*F. tinnunculus*) in the surveyed area, perched on TV antennas. We never recorded any instances of predation or competition between the species detected. While feral pigeons could be considered scavengers in the city, on the studied roofs they primarily fed on the available vegetation resources. Breeding or reproductive behaviour was not detected like in other green roofs studied [33,34]. This can be attributed to the lack of suitable habitat and enough surface, but in any case, the species observed in our green roof are not ground nesters as the breeding birds recorded in other studies [25], but cavity nesters or tree nesters. Nevertheless, the blackbirds (*T. merula*) bred in a close garden, and during the spring, they were observed foraging on the green roof to provide their offspring. In this sense, the green roof can provide food for breeding birds although it does not act as breeding territory.

In conclusion, we show how a green roof located in the core of a major European city is used occasionally by different birds in all the seasons, mainly for feeding, with higher species richness in the spring and less daily use in the first hours after the sunrise. We consider that the plantation of more species than *Sedum*, with different phenology and traits, could contribute to creating a more heterogeneous green roof, favouring a higher use of the birds present in the area by increasing the diversity of food throughout the year [54]. In fact, although we did not measure it directly, we observed the appearance of spontaneous plant species during the warmer seasons, so it would be interesting to investigate the relationship of these plants and the bird richness in a future study. Moreover, the creation of this green roof could act as a starting point to replicate similar sustainable ideas in this and other cities, helping to increase the total surface of green areas and facilitating the connection between parks by creating stepping stones. This gains in interest when we consider the high and low temperatures of the city in the summer and the winter, respectively, the low number of green areas in some neighbourhoods and the current context of climate change. Further research is needed to evaluate the presence of nocturnal birds, and permanent monitoring could offer additional information regarding patterns across years. Last, future studies could replicate this research across multiple green roofs in different cities and countries, with varying heights and management strategies, to better understand the relative roles of roof elevation and maintenance practices in shaping bird communities.

## Data Availability

Link to repository: [55]. Supplementary material is available online [56].
